# Altered plasma arginine metabolome precedes behavioural and brain arginine metabolomic profile changes in the APPswe/PS1ΔE9 mouse model of Alzheimer’s disease

**DOI:** 10.1038/s41398-018-0149-z

**Published:** 2018-05-25

**Authors:** DH Bergin, Y Jing, BG Mockett, H Zhang, WC Abraham, P Liu

**Affiliations:** 1 Department of Anatomy, University of Otago, Dunedin, New Zealand; 2 School of Pharmacy, University of Otago, Dunedin, New Zealand; 3 Brain Research New Zealand and Brain Health Research Centre, University of Otago, Dunedin, New Zealand; 4 Department of Psychology, University of Otago, Dunedin, New Zealand

## Abstract

While amyloid-beta (Aβ) peptides play a central role in the development of Alzheimer’s disease (AD), recent evidence also implicates altered metabolism of L-arginine in the pathogenesis of AD. The present study systematically investigated how behavioural function and the brain and plasma arginine metabolic profiles changed in a chronic Aβ accumulation model using male APPswe/PS1ΔE9 transgenic (Tg) mice at 7 and 13 months of age. As compared to their wild-type (WT) littermates, Tg mice displayed age-related deficits in spatial water maze tasks and alterations in brain arginine metabolism. Interestingly, the plasma arginine metabolic profile was markedly altered in 7-month Tg mice prior to major behavioural impairment. Receiver operating characteristic curve analysis revealed that plasma putrescine and spermine significantly differentiated between Tg and WT mice. These results demonstrate the parallel development of altered brain arginine metabolism and behavioural deficits in Tg mice. The altered plasma arginine metabolic profile that preceded the behavioural and brain profile changes suggests that there may be merit in an arginine-centric set of ante-mortem biomarkers for AD.

## Introduction

Alzheimer’s disease (AD), the most common form of dementia, is characterised by the histopathological features of hyperphosphorylated tau and accumulations of amyloid-beta (Aβ) that form neurofibrillary tangles and amyloid plaques in the brain, respectively^1,2^. The amyloid cascade hypothesis proposes that increased levels of pathogenic Aβ peptides derived from proteolytic cleavage of amyloid precursor protein (APP) are critical to the aetiology of AD^1,3^. While this hypothesis has been leading the field for over two decades, the causality of Aβ has been increasingly challenged^4^, and other neuropathological features of AD are gathering increasing interest. Recent research has suggested that altered metabolism of L-arginine may be involved in the pathogenesis and development of AD^5–11^.

L-arginine is metabolised to a number of bioactive molecules in mammals in a tightly regulated manner. The dominant L-arginine metabolic pathway is through nitric oxide (NO) synthase (NOS), which generates the gaseous signalling molecule NO^12^. NO derived from endothelial NOS (eNOS) is essential for maintaining normal cerebral blood flow^5,13^. Earlier research demonstrated a close association between plaques, tangles and reduced capillary eNOS expression in AD brains^14,15^. Recently, we found markedly reduced eNOS protein expression in the superior frontal gyrus and hippocampus (two brain regions that are affected early and severely in the disease) of AD cases when compared to their age- and sex-matched control cases^8^. Experimentally, eNOS-deficient (eNOS^−^^/−^) mice display memory deficits and increased levels of APP, β-site APP-cleaving enzyme 1 (BACE1), Aβ, and neuroinflammation in the brain^16^, indicating a contribution of the loss of eNOS-derived NO to amyloidogenic processing of APP and cognitive decline. In addition to its role as a vasodilator, NO derived from neuronal NOS (nNOS) plays an important role in synaptic plasticity and learning and memory^17–19^, while that from inducible NOS (iNOS) acts as a proinflammatory agent^7,20^. Aberrant NO synthesis, particularly that derived from iNOS, leads to neurotoxicity and neurodegeneration^7,20–24^. We found marked AD-related decreases in nNOS expression, but intensively labelled iNOS-immunoreactive neurons and astrocytic clusters in the superior frontal cortex of AD cases^8^. This latter finding may indicate the deleterious presence of iNOS in the tangle-bearing neurons and astrocytes surrounding the plaques.

L-arginine can also be metabolised by arginase and arginine decarboxylase to generate L-ornithine and agmatine, respectively^12,25^. L-ornithine can be further metabolised to form polyamines putrescine, spermidine and spermine that are essential for normal cell growth and function, or via a separate pathway to form glutamate and γ-aminobutyric acid (GABA)^12^. Agmatine is a putative neurotransmitter that contributes to learning and memory processes and plays an important role in regulating the production of NO and polyamines^25–28^. We found AD-related increases in arginase activity and arginase II protein expression in the superior frontal cortex, hippocampus and cerebellum, but decreases in L-ornithine (the product of arginase), agmatine and polyamines in a region-specific manner^8^. Inoue et al., however, reported increased polyamine levels in the AD frontal and occipital lobes^6^.

In summary, human AD post-mortem brain tissue research has revealed alterations in the levels of L-arginine and its metabolites in areas highly affected by the disease, suggesting their possible contribution to the neuropathology and cognitive impairments of the disease^6,8^. It remains a question, however, how early in the disease process that alterations in the L-arginine metabolome begin to occur. The use of animal models has begun to reveal relationships between Aβ and arginine metabolism in the brain. For example, we have demonstrated that a single intracerebroventricular injection of Aβ_25–35_ (the proposed toxic domain of Aβ) alters L-arginine metabolism in the rat prefrontal cortex and hippocampus at 8, 42 and 97 days post infusion^29,30^. In another model of AD, APPswe/PSEN1ΔE9 (APP/PS1) transgenic mice display progressive brain Aβ accumulation and behavioural impairments from 4 months of age, which become progressively apparent up to and beyond 12 months of age^31–35^. We took advantage of this progressive model to determine the behavioural and neurochemical changes in response to chronic cerebral Aβ accumulation at different pathological stages. We predicted that APP/PS1 mice would show progressive cognitive deficits in association with altered brain arginine metabolism, as seen in AD patients^8^. To test this hypothesis, we assessed spatial learning and memory in male APPswe/PS1ΔE9 mice using the water maze task, and systematically determined the arginine metabolic profile in the brain at 7 and 13 months of age.

Peripheral blood has been increasingly used to identify biomarkers of brain pathology^36,37^. To this end, we also analysed the arginine metabolic profiles in the blood (plasma) from the same animals, aiming to compare the brain and plasma profile changes both in terms of the direction and degree of changes, as well as their time course.

## Materials and methods

### Animals

Male B6C3-Tg(APPswe,PSEN1ΔE9)85Dbo/Mmjax^38,39^ and B6C3 wild-type (WT) females were originally obtained from Jackson Laboratories (http://jaxmice.jax.org/strain/004462.html) and crossed, resulting in hemizygote transgenic (Tg) and WT littermates. Seven-month-old (WT, *n* = 9; Tg, *n* = 9; from six litters) and thirteen-month-old (WT, *n* = 14; Tg, *n* = 15; from 12 litters) animals were housed individually (13 × 15 × 38 cm^3^), maintained on a 12-h light/dark cycle (lights on at 8 AM) and provided ad libitum access to food and water. The sample size was based on a pilot study followed by a power calculation. All experimental procedures were carried out in accordance with the regulations of the University of Otago Committee on Ethics in the Care and Use of Laboratory Animals and New Zealand legislation. Every attempt was made to limit the numbers of animals used and to minimise suffering.

### Behavioural procedures

Behavioural testing was conducted in a windowless room with a video camera mounted at ceiling height in the centre of the room and four 60 W bulbs mounted on the ceiling in the corners of the room. There was a radio speaker adjacent to the video camera to provide background-masking noise. The extramaze cues (the laboratory furniture, lights and several prominent visual features on the walls, as well as the location of the experimenter) were held constant throughout the entire study.

The water maze pool was a white plastic circular tank measuring 100 cm in diameter and 35 cm in height. It was filled daily to 15 cm below the top and maintained at 20 ± 1 °C. Four points around the edge of the pool were designated as north (N), south (S), east (E) and west (W), which allowed the pool to be divided into four quadrants (i.e., NE, SW, NW and SE). Behavioural procedures were based on our water maze protocols in rats^40,41^. For each trial, the mouse was placed into the pool facing towards the wall and allowed to swim in search of the platform for a maximum of 60 s. Mice were permitted to stay on the platform for 10 s before being removed, dried and placed into a holding box. If the mouse did not find the platform within 60 s of being placed into the pool, it was immediately placed on or if near guided to the platform for 10 s before being returned to the holding box. Starting locations (N, S, W and E) were pseudorandomly selected and kept the same for all of the animals.

#### Cued navigation (days 1 and 2)

A clear platform (6 cm in diameter) was raised 0.5 cm above the water surface and located in the centre of the maze. An upright plastic tag 5 × 3 cm was attached to the edge of the platform to make it more visible. There were four trials on the first day and five trials on the second day with 60-s intertrial intervals.

#### Reference memory version (days 3–7)

Animals were trained to find the hidden platform (0.5 cm below the water surface) located in the centre of the SE quadrant for 5 consecutive days with six trials each day and 60-s intertrial intervals.

After completion of the place navigation training, the platform was removed from the pool, and a probe trial was conducted either 2 min (for 7-month-old mice) or 24 h (for 13-month-old mice) after the final training trial. All animals were placed into the pool from a fixed starting point (N) and allowed to swim freely for 60 s.

#### Working memory version (days 8–10)

After reference memory testing, mice were tested in the working memory version of the water maze task from day 8 for 3 consecutive days. The platform location was changed each day, but was kept the same for both genotype groups across all seven trials. During the first six trials, animals were trained to find a visible platform (trial 1) followed by a hidden platform placed in the same location (trials 2–6). There were 60-s intertrial intervals and the maximum time allowed to search for the platform was 60 s. Mice were allowed to remain on the platform for 10 s before being removed, dried and placed into their homecages. Trial 7 was a 60-s probe trial where the platform was removed from the pool, and was conducted 120 s following the six trial. The starting locations were different for each trial, but were kept the same for all of the animals.

#### Measured variables

Following completion of the water maze tasks, several performance variables were analysed from HVS 2020 (HVS Image Software Ltd, Bicester, UK), as described in our previous rat studies^40,41^. The distance the mice swam from the starting point to reach the platform (path length) and the degree of thigmotaxic swimming (i.e., swimming close to the wall) were measured. Thigmotaxic swimming was quantified by dividing the maze into two circles and measuring the time spent in the outer 10% of the pool. For the probe trials, the path length to the first platform crossing, the percentage of time spent in the target quadrant and the number of crossings over the previous platform location were determined.

### Neurochemical procedures

#### Blood and brain tissue preparation

One week after the final day of behavioural testing, all mice were killed by decapitation without anaesthesia. For each animal, trunk blood was collected at the time of decapitation in an EDTA-coated tube and stored on ice. The EDTA tubes were centrifuged at 2000×*g* for 10 min at 4 °C (Eppendorf 5810) and the plasma was then collected. Ice-cold ethanol/methanol (50/50; v/v) was added to each plasma sample at a 4:1 ratio, and the mixture was centrifuged at 12,000×*g* for 10 min at 4 °C. The supernatant from each tube was stored at −80 °C until the high-performance liquid chromatography (HPLC) and liquid chromatography/mass spectrometric (LC/MS) assays.

The brain from each animal was rapidly removed and transferred to cold saline for at least 45 s. The prefrontal cortex (PFC), whole hippocampus (HPC), parahippocampal region (PH, containing the entorhinal, perirhinal and postrhinal cortices) and cerebellum (CE) were dissected freshly on ice from each hemisphere. Procedures for the dissections were based on the studies of Hortnagl et al. and Burwell et al.^42,43^ and our previous rat studies^44–48^. The brain tissue samples harvested from one hemisphere were snap-frozen and stored at −80 °C until the enzyme assays and western blotting were carried out. The samples harvested from the other hemisphere were weighed, homogenised in ice-cold 10% perchloric acid (~50 mg wet weight per ml) and centrifuged at 10,000×*g* for 10 min to precipitate protein. The perchloric acid extracts (supernatants) were then stored at −80 °C until the HPLC and LC/MS assays.

#### NOS and arginase assays

At the time of the protein assay, protease-inhibitory buffer (containing 50 mM Tris-HCl (pH 7.4), 10 μM phenylmethylsulfonyl fluoride, 15 μM pepstatin A and 2 μM leupeptin) was added to each brain tissue sample on ice. The samples were then homogenised and centrifuged at 12,000×*g* for 10 min at 4 °C. Protein concentration in the supernatant was determined using the Bradford method^49^. Each supernatant was then separated into three parts and used for the NOS and arginase assays and western blot, respectively.

Radioenzymatic and spectrophotometric assays were used to determine total NOS and arginase activities by measuring the ability of tissue homogenates to convert [^3^H] L-arginine to [^3^H] L-citrulline in the presence of cofactors, and the amount of newly formed urea from L-arginine, respectively, as described previously^44–48,50–57^. The contribution of iNOS (calcium-independent) to total NOS activity was assessed in the absence of calcium. All assays were performed in duplicate. For each brain region, the tissues from both the WT and Tg groups were processed at the same time in a counterbalanced manner. The experimenters were blind to the grouping information. NOS and arginase activities were expressed as pmol [^3^H] L-citrulline/30 min/mg protein and μg urea/mg protein, respectively.

#### Western blotting

The protein expression of nNOS, eNOS, arginase I, arginase II and β-actin in each brain tissue sample was determined using western blots. The protein concentrations in all of the brain tissue samples were equalised to 2 mg/ml. Brain tissue homogenates were then mixed with gel-loading buffer (containing 50 mM Tris-HCl, 10% SDS, 10% glycerol, 10% 2-mercaptoethanol and 2 mg/ml bromophenol blue) in a ratio of 1:1 and then boiled for 5 min. Eight microlitres of each sample were loaded in each well on a Bis-Tris Criterion gel (Bio-Rad), and a pre-stained protein marker (10–250 kDa; Bio-Rad) was run on the same gel. The proteins were then transferred overnight to polyvinylidene-difluoride membranes. Pre-stained protein markers (Bio-Rad) were always run on the same gel. The membranes were blocked by incubation with 5% dried milk protein and 0.1% BSA for 6 h, drained and then incubated with primary polyclonal rabbit antibodies raised against eNOS (1:7500, sc-653) or arginase II (1:1500, sc-2015) or monoclonal mouse antibodies raised against nNOS (1:7500, sc-5302), arginase I (1:1000, sc-166920) or β-actin (1:200,000, sc-47778) overnight at 4 °C. The secondary antibody was anti-rabbit IgG (sc-2004) or anti-mouse (sc-2005) linked to horseradish peroxidase. Detection was performed using the enhanced chemiluminescence system (Amersham, New Zealand). Hyperfilms were analysed by densitometry using the Bio-Rad Quantity One software. Results were expressed as volume of the band (optical density × area of the band), and normalised by the corresponding β-actin loading controls^8,51,58^.

#### Amino-acid and polyamine analyses

The brain levels of amino acids (L-arginine, L-citrulline, L-ornithine, glutamate and GABA) and the polyamines spermidine and spermine were quantified by HPLC, while agmatine and putrescine levels were measured by a highly sensitive LC/MS/MS method, as we have previously described^8,44–47,50,51^. High-purity external and internal standards were used (Sigma, Sydney, Australia). All other chemicals were of analytical grade. The samples from both the WT and Tg groups at one age point were assayed at the same time in a counterbalanced manner, and the experimenters were blind to the grouping information. The assays were performed in duplicate. The concentrations of L-arginine and its eight downstream metabolites in tissue were calculated with reference to the peak area of external standards, and values were expressed as μg/g wet tissue^8,44–47,50,51^.

To determine the plasma levels of L-arginine and its downstream metabolites, the plasma/ethanol/methanol supernatant was divided into two parts for the measurements of amino acids (L-arginine, L-citrulline, L-ornithine, glutamate and GABA) and amines (agmatine, putrescine, spermidine and spermine) using the HPLC or LC/MS assays, respectively. Both assays were conducted in duplicate. Both the samples from WT and Tg mice at 7 and 13 months of age were assayed at the same time in a counterbalanced manner and the experimenters were blind to the grouping information. The concentrations of six amino acids and four amines in plasma were calculated with reference to the peak area of external standards, and values were expressed as nmol/ml plasma and pmol/ml plasma, respectively.

### Statistical analysis

Behavioural data were analysed using two-way analysis of variance (ANOVA) followed by Bonferroni post hoc tests and/or Student’s *t*-tests. Because brain neurochemicals were assayed separately at each age point, Student’s *t*-tests were used to determine the effects of genotype. Plasma data were analysed using two-way ANOVA followed by Bonferroni post hoc tests, as the samples from both age groups were assayed at the same time. Statistical analysis was performed using Graphpad Prism software, and all data were presented as mean ± SEM. The level of significance was set at *p* ≤ 0.05 for all comparisons^59,60^.

In order to determine the capacity of the plasma levels of L-arginine metabolites to predict which animals are APP/PS1 mice, receiver operating characteristic (ROC) curve analysis was performed, and the sensitivity and specificity, and positive and negative predictive values were obtained.

Finally, correlational analyses were conducted to determine the relationships between brain and blood neurochemical variables, and the level of significance was set at 0.01 (equivalent to a Geisser–Greenhouse correction for potential violation of the assumption of sphericity^60^).

## Results

### Body weight

There were no significant differences between the WT and their Tg littermates at 7 (WT: 42 ± 1.4 g; Tg: 41 ± 1.2 g; *t* < 1) and 13 (WT: 41 ± 1.3 g; Tg: 43 ± 1.1 g; *t* < 1) months of age, when weighed on the day of their killing.

### Behavioural data

#### Cued navigation

**Fig. 1:**
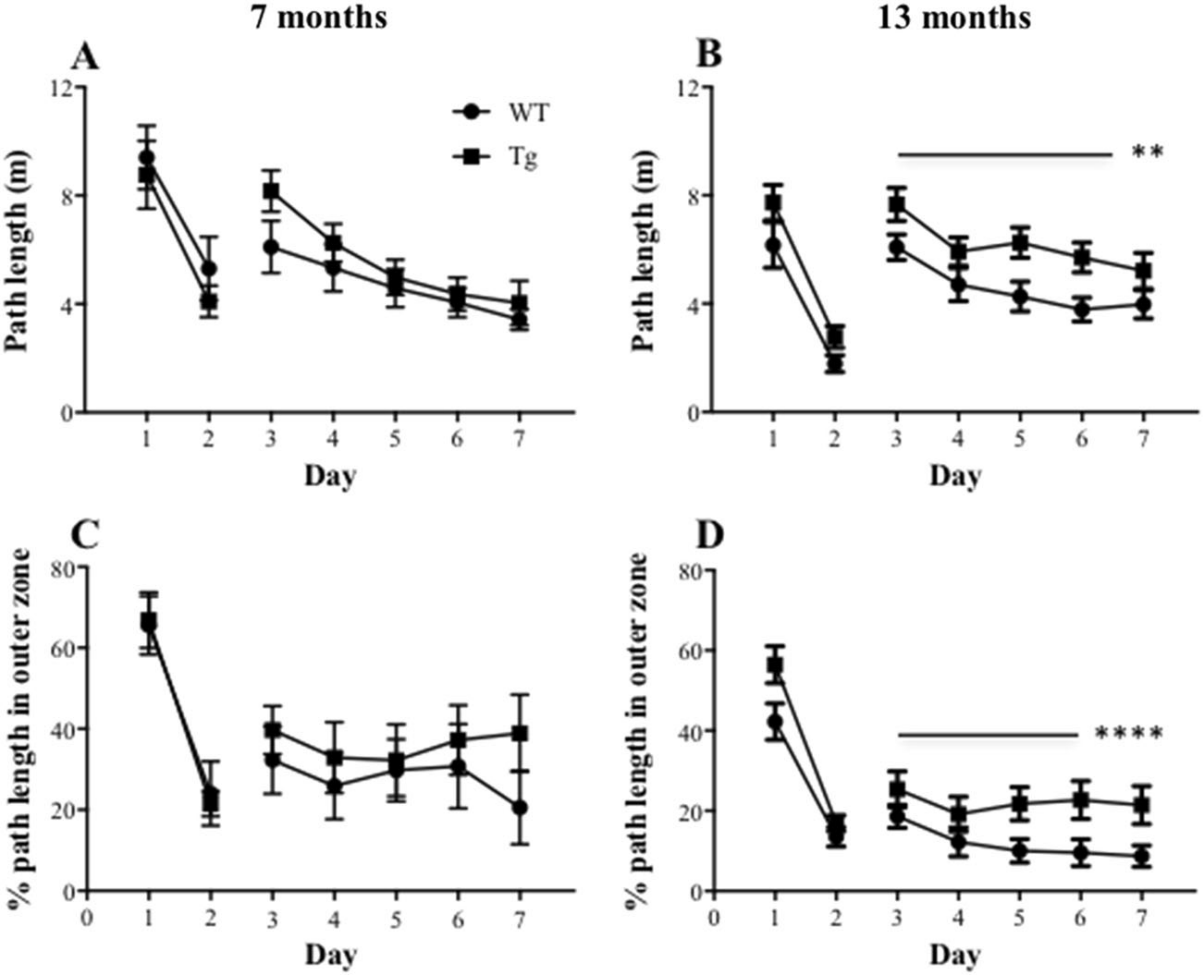
Animals’ performance in the reference memory version of the water maze task: APPswe/PS1ΔE9 transgenic (Tg) mice at 13 months of age generated longer path length to reach the platform during the place navigation relative to wild-type (WT) mice. Mean (±SEM) path length (m) to reach the platform (**a**, **b**) and percentage of path length spent in the outer 10% of the pool (thigmotaxic swimming; **c**, **d**) during the cued (days 1 and 2) and place (days 3–7) navigation in the WT and Tg mice at 7 (**a**, **c**) and 13 (**b**, **d**) months of age. * indicates a significant effect of day at **p < 0.01, ****p < 0.0001

For cued navigation by the 7-month-age groups, two-way ANOVA revealed significant main effects of day for both path length (*F*(1,16) = 25.97, *P* = 0.0001; Fig. 1a) and thigmotaxis (*F*(1,16) = 66.95, *P* < 0.0001; Fig. 1c), with markedly reduced path lengths and thigmotaxis on day 2 relative to day 1. For the 13-month-age groups, there were again significant main effects of day for path length (*F*(1,27) = 113.94, *P* < 0.0001; Fig. 1b) and thigmotaxis (*F*(1,27) = 125.39, *P* < 0.0001; Fig. 1d), again with improved performance on the second day of testing. However, there were no significant effects of genotype or genotype × day interactions for either variable for either age group (all *F* < 1). These findings indicate that all groups were capable of learning this cued version of the water maze task.

#### Spatial reference memory

For the spatial reference memory task, the 7-month-age groups showed significantly reduced path lengths across 5 days of training for both genotypes (*F*(4,64) = 11.84, *P* < 0.0001; Fig. 1a), but no effect of genotype (*F*(1,64) = 1.70, *P* = 0.21). However, there were no significant effects of genotype, day or their interaction for thigmotaxis (all *F* ≤ 1; Fig. 1c). In contrast, for the 13-month-age groups, there were significant main effects of genotype (path length: *F*(1,27) = 8.47, *P* = 0.007; thigmotaxic swimming: *F*(1,27) = 4.35, *P* = 0.05) and day (path length: *F*(4108) = 8.03, *P* < 0.0001; thigmotaxic swimming: *F*(4108) = 3.89, *P* = 0.005), but not their interaction (all *F* < 1), with greater path lengths and more thigmotaxic swimming in Tg mice compared to WT mice (Fig. 1b,d). Collectively, these results indicate age-related deficits in spatial learning in the APP/PS1 mice.

To assess animals’ memory for the platform location, a probe test was conducted 2 min (for the 7-month groups) or 24 h (for the 13-month groups, to make the recall task harder) after the final training trial. For both age groups, there were no significant genotype differences in the path length to the first platform crossing, the number of platform crossings or the percentage of time spent in the target quadrant (all *t* ≤ 1; Supplementary Fig. 1A-1F).

#### Spatial working memory

**Fig. 2:**
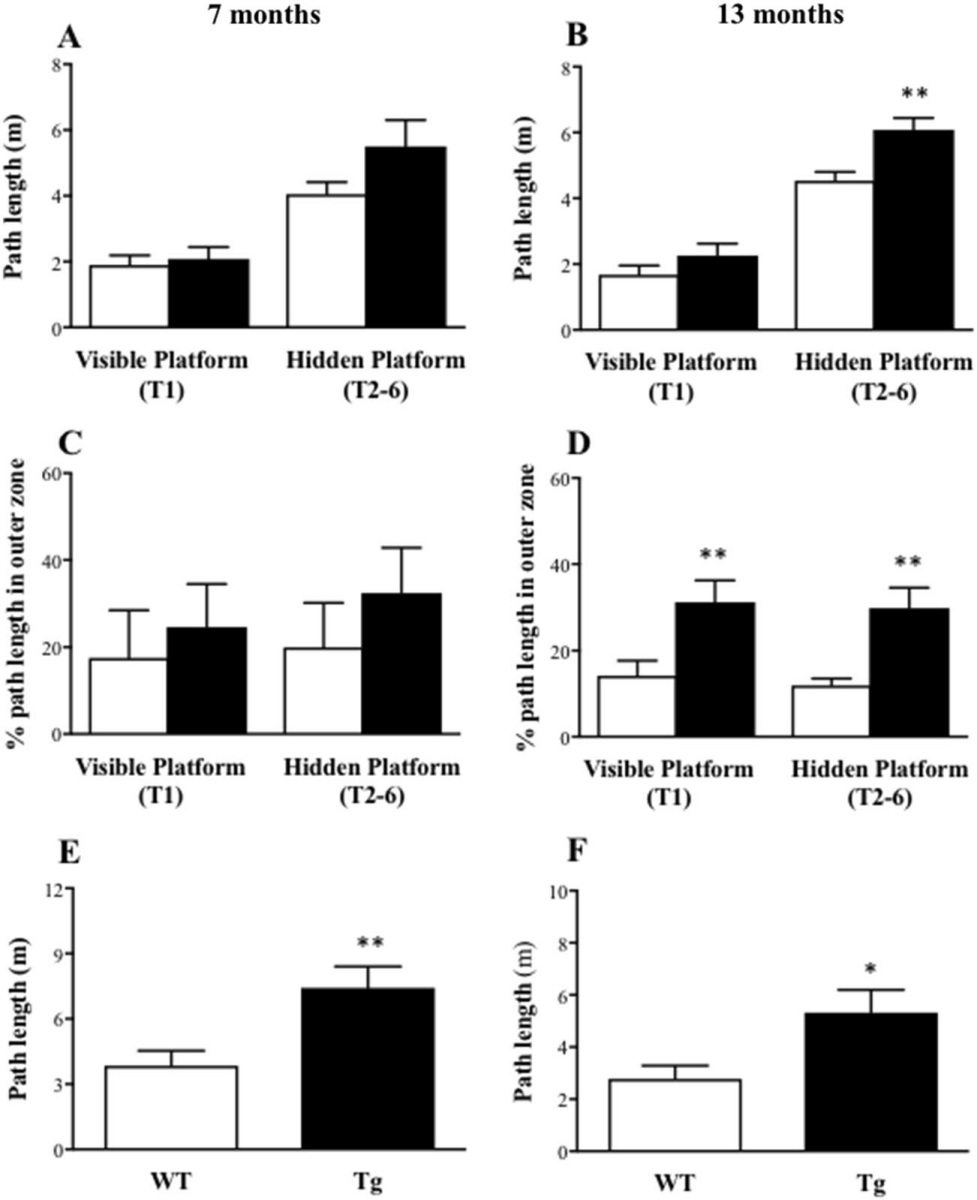
Animals’ performance in the working memory version of the water maze task: APPswe/PS1ΔE9 transgenic (Tg) mice generated longer path length and greater percentage of thigmotaxic swimming to reach the platform (for the 13-month group) and greater path length to the platform location during the probe test (for both age groups) relative to their age-matched wild-type (WT) mice. Mean (±SEM) path length (m) to reach the platform (**a**, **b**) and percentage of path length spent in the outer 10% (thigmotaxic swimming; **c**, **d**) during trial 1 (T1; visible platform) and trials 2–6 (T2–6; hidden platform), and path length to the platform location (**e**, **f**) during trial 7 (probe test) in the WT and Tg mice at 7 (**a**, **c**, **e**) and 13 (**b**, **d**, **f**) months of age. * indicates a significant difference between genotypes at *p < 0.05, **p < 0.01

All animals were tested in the spatial working memory version of the water maze task for 3 days, and the first day was treated as habituation. The behavioural data generated on trial 1 (visible platform), trials 2–6 (hidden platform) and trial 7 (probe test) over the last 2 days were separately averaged. For the 7-month-age groups, there were no significant differences between WT and Tg mice in either path length (Fig. 2a) or thigmotaxis (Fig. 2c) when searching for the visible or hidden platform. For the 13-month age groups, however, Tg mice generated significantly greater path lengths to reach the hidden platform (*t*(27) = 3.43, *P* = 0.002), but not the visible platform (*t* < 1) relative to WT mice (Fig. 2b). Tg mice also generated greater thigmotaxic swimming when searching for the visible (*t*(27) = 2.77, *P* = 0.01) and hidden (*t*(27) = 3.56, *P* = 0.001) platforms compared to WT mice (Fig. 2d). During the probe test, Tg mice generated greater path lengths to reach the previous platform location when compared to WT mice at both 7 (*t*(16) = 2.99, *P* = 0.009; Fig. 2e) and 13 (*t*(27) = 2.47, *P* = 0.02; Fig. 2f) months of age, with no significant genotype differences in terms of the number of platform crossings and the percentage of time spent in the target quadrant (all *t* < 1; Supplementary Fig. 1G-1J). Overall, these results showed a progressive age-related impairment in spatial learning and memory abilities in the Tg mice compared to the WT mice.

### Neurochemical data

#### NOS and arginase activity and protein expression

Radioenzymatic and spectrophotometric assays and western blotting revealed no genotype differences in total NOS and arginase activities, or the protein levels of nNOS, eNOS and arginase II, in either the prefrontal cortex, hippocampus, parahippocampal region or cerebellum at 7 months of age. However, there was a lower level of arginase I protein expression in the Tg prefrontal cortex (*t*(16) = 2.58, *P* = 0.02; 43% decrease, Supplementary Fig. 2). For the 13-month-age groups, there were no significant genotype differences in nNOS activity or in eNOS, arginase I and arginase II protein expression in any region examined. There was a 17% reduction in arginase activity in the Tg parahippocampal region (*t*(24) = 2.88, *P* = 0.008) and 37% higher levels of nNOS protein expression in the Tg prefrontal cortex (*t*(22) = 3.12, *P* = 0.005; Supplementary Fig. 2). iNOS activity was not detectable in any of the brain regions of WT and Tg mice, at either age.

#### Brain profiles of L-arginine and its downstream metabolites

**Fig. 3:**
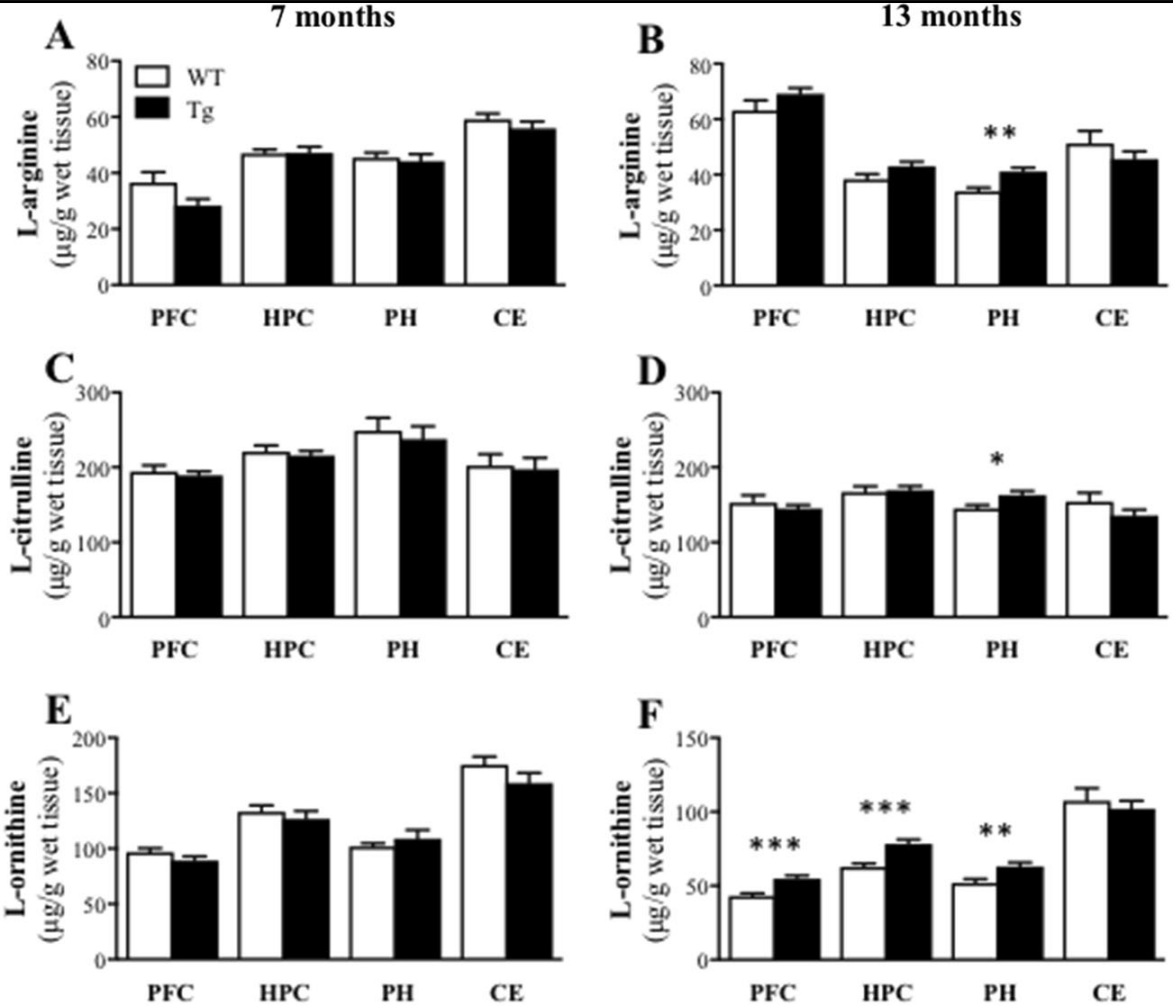
Brain amino-acid profile changes: there were increased L-arginine, L-citrulline and L-ornithine levels in the prefrontal cortex (PFC), hippocampus (HPC), parahippocampal region (PH) in APPswe/PS1ΔE9 transgenic (Tg) mice at 13 months of age relative to the wild-type (WT) mice. Mean (±SEM) L-arginine (**a**, **b**), L-citrulline (**c**, **d**) and L-ornithine (**e**, **f**) levels in the PFC, HPC, PH and cerebellum (CE) of the WT and Tg mice at 7 (**a**, **c**, **e**) and 13 (**b**, **d**, **f**) months of age. * indicates significant difference between genotypes at *p < 0.05, **p < 0.01, ***p < 0.001

**Fig. 4:**
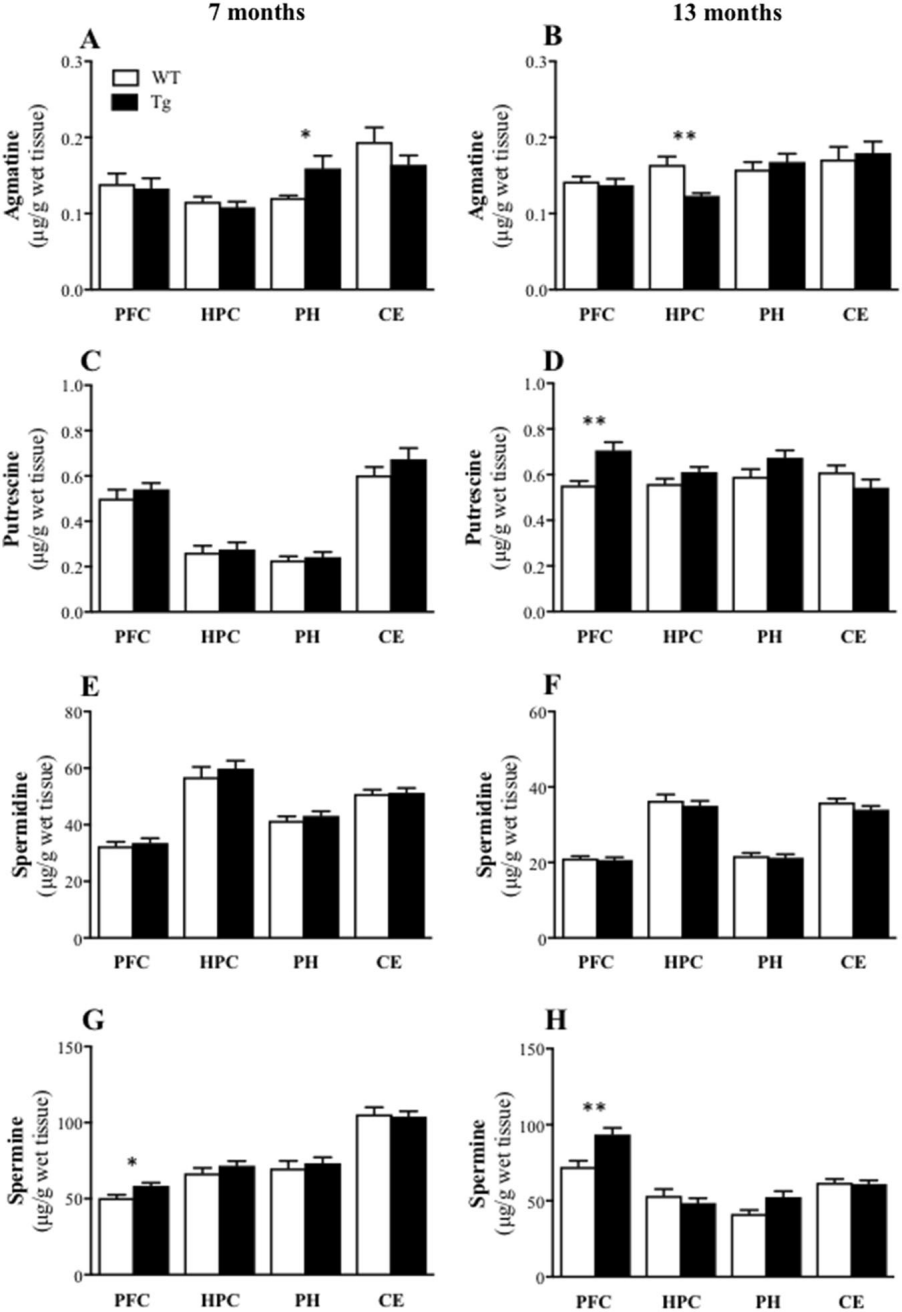
Brain amine profile changes: APPswe/PS1ΔE9 transgenic (Tg) mice had increased levels of agmatine in the parahippocampal region (PH) and spermine in the prefrontal cortex (PFC; for the 7-month group), and reduced agmatine levels in the hippocampus (HPC) but increased levels of putrescine and spermine in the PFC (for the 13-month group) relative to the wild-type (WT) mice. Mean (±SEM) agmatine (**a**, **b**), putrescine (**c**, **d**), spermidine (**e**, **f**) and spermine (**g**, **h**) levels in the PFC, HPC, PH and cerebellum (CE) of the WT and Tg mice at 7 (**a**, **c**, **e**, **g**) and 13 (**b**, **d**, **f**, **h**) months of age. * indicates significant difference between genotypes at *p < 0.05, **p < 0.01

There were no significant genotype differences in L-arginine (Fig. 3a), L-citrulline (Fig. 3c) or L-ornithine (Fig. 3e) in any brain region examined in 7-month old mice (all *t* ≤ 1). At 13 months, however, Tg mice had significantly higher levels of L-arginine (*t*(27) = 3.57, *P* = 0.002; 21% increase, Fig. 3b), L-citrulline (*t*(27) = 2.72, *P* *=* 0.011; 12% increase, Fig. 3d) and L-ornithine (*t*(27) = 2.98, *P* = 0.006; 21% increase, Fig. 3f) in the parahippocampal region relative to WT mice. Moreover, there were increased levels of L-ornithine in the prefrontal cortex (*t*(27) = 4.02, *P* = 0.0004; 28% increase) and hippocampus (*t*(27) = 3.98, *P* = 0.0005; 26% increase) in Tg mice (Fig. 4f). Glutamate and GABA concentrations were similar between WT and Tg mice at both ages for all brain regions examined (all *t* ≤ 1; data not shown).

For agmatine and the downstream polyamines, there were higher levels of agmatine in the parahippocampal region (*t*(15) = 2.45, *P* = 0.027; 33% increase, Fig. 4a) and spermine in the prefrontal cortex (*t*(16) = 2.75, *P* = 0.014; 17% increase, Fig. 4g) in Tg mice relative to WT mice at 7 months of age. For the 13-month-age groups, in contrast, agmatine was significantly decreased in the Tg hippocampus (*t*(27) = 3.77, *P* = 0.001; 27% decrease, Fig. 4b). There were also increased putrescine (*t*(27) = 3.66, *P* = 0.0011; 29% increase, Fig. 4d) and spermine (*t*(27) = 3.58, *P* = 0.0013; 29% increase, Fig. 4h) levels in the Tg prefrontal cortex, with no significant genotype differences for spermidine in any brain region at either age (all *t* ≤ 1; Fig. 4e,f).

#### Plasma profiles of L-arginine and its downstream metabolites

**Fig. 5:**
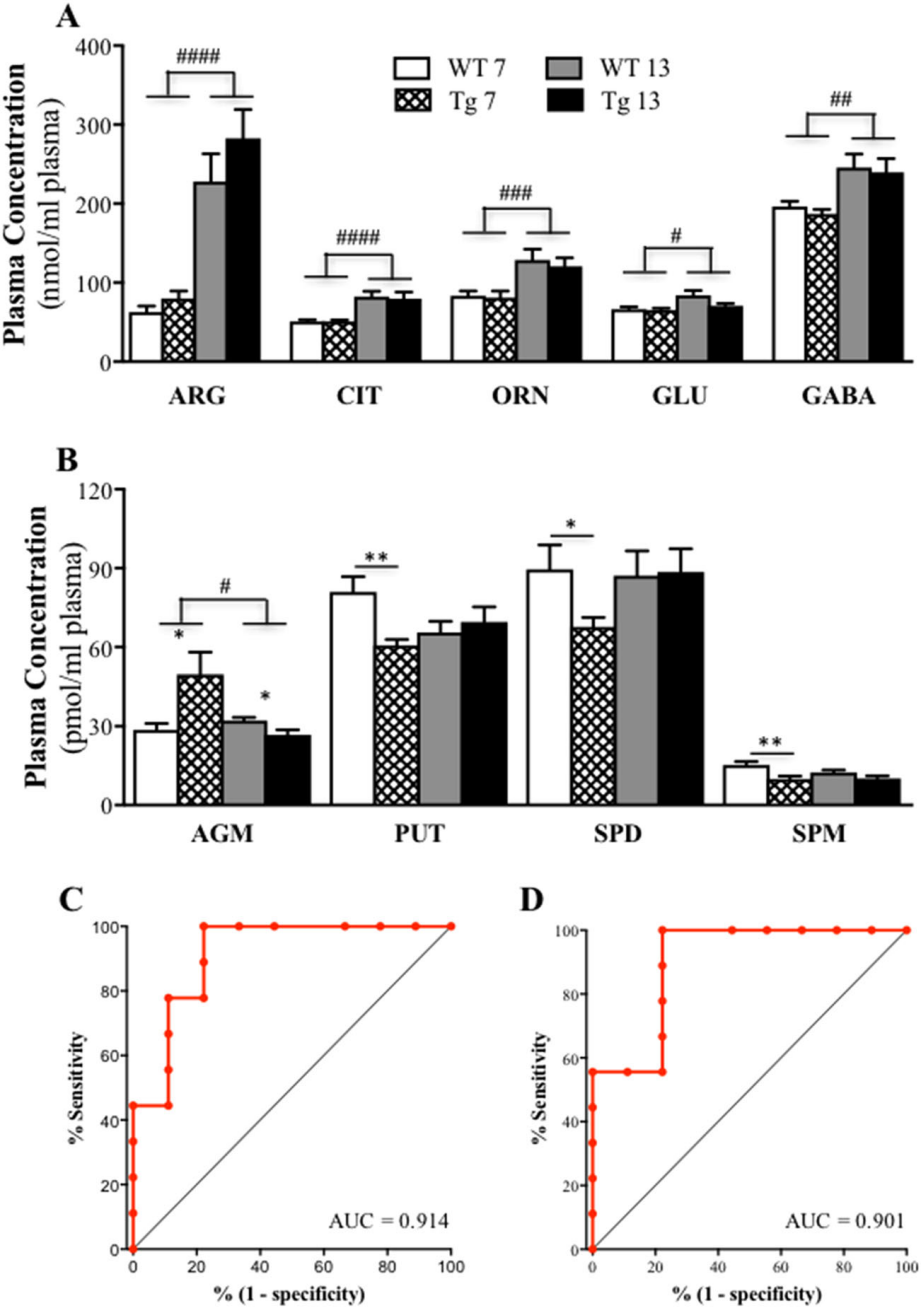
Plasma arginine metabolic profile changes. Both wild-type (WT) and APPswe/PS1ΔE9 transgenic (Tg) mice displayed age-related increases in the plasma L-arginine (ARG), L-citrulline (CIT), L-ornithine (ORN), glutamate (GLU) and γ-aminobutyric acid (GABA) levels. Tg mice had higher agmatine (AGM) level but lower putrescine (PUT), spermidine (SPD) and spermine (SPM) levels at 7 months of age, and slightly reduced AGM level at 13 months of age relative to their age-matched WT controls. Mean (±SEM) ARG, CIT, ORN, GLU and GABA (**a**), and AGM, PUT, SPD and SPM (**b**) levels in the plasma of the WT and Tg mice at 7 and 13 months of age, and the receiver-operator characteristic (ROC) curves for plasma putrescine (**c**) and spermine (**d**) levels in Tg mice and their age-matched WT littermates at 7 months of age (AUC, area under the curve). * indicates significant difference between WT and Tg mice at *p < 0.05, **p < 0.01. # indicates significant difference between 7- and 13-month-old mice at ^#^*p* < 0.05, ^##^*p* < 0.01, ^###^*p* < 0.001, ^####^*p* < 0.0001

We also determined whether changes in the plasma arginine metabolome mirrored those in the brain. For the five amino acids (Fig. 5a), two-way ANOVA revealed that the 13-month-old WT and Tg mice had higher concentrations of L-arginine (*F*(1,43) = 32.04, *P* < 0.0001; 72% increase), L-citrulline (*F*(1,43) = 18.93, *P* < 0.0001; 37% increase), L-ornithine (*F*(1,43) = 13.46, *P* = 0.0007; 34% increase), glutamate (*F*(1,43) = 6.82, *P* = 0.012; 15% increase) and GABA (*F*(1,43) = 10.20, *P* = 0.003; 21% increase) when compared to the 7-month-old mice, with no significant effects of genotype or genotype × age interaction (all *P* > 0.05).

The plasma levels of agmatine and polyamines in WT and Tg mice at both 7 and 13 months of age are presented in Fig. 5b. For agmatine, there were significant effects of genotype (*F*(1,43) = 4.45, *P* *=* 0.04), age (*F*(1,43) = 6.83, *P* = 0.012) and their interaction (*F*(1,43) = 12.81, *P* = 0.0009). Bonferroni post hoc tests revealed that these effects were largely due to the Tg mice having a higher level of agmatine at 7 months of age (*P* < 0.05; 73% increase), but a lower level at 13 months of age (*P* < 0.05; 18% decrease). For putrescine, there was a significant genotype × age interaction (*F*(1,43) = 5.38, *P* = 0.025), but no effect of genotype (*F*(1,43) = 2.44, *P* = 0.13) or age (*F* < 1*)*, and the post hoc test indicated a significant reduction (25%) in Tg mice relative to WT mice at 7 months of age (*P* < 0.01). For spermidine, there were no significant effects of genotype, age or their interation. A planned comparison, however, revealed a 25% decrease in spermidine in Tg mice relative to WT mice at 7 months of age (*t*(16) = 2.21, *P* = 0.04). For spermine, there was a significant genotype difference (*F*(1,43) = 12.07, *P* = 0.0012), with a 35% reduction in Tg mice relative to WT mice at 7 months of age (*P* < 0.01).

The above results demonstrated that plasma levels of agmatine showed a progressive decline with age, while the three polyamines were reduced in the Tg mice at 7 but not 13 months of age. In order to determine the capacity of the four amines in plasma to predict the genotype of animals, a ROC curve analysis was performed. For putrescine, interestingly, the area under the curve (AUC) was calculated as 0.914 (95% confidence interval, 0.69–0.99 *P* < 0.0001), with the sensitivity and specificity values being 100% and 78%, respectively (Fig. 5c). Similarly, plasma spermine had an AUC of 0.901 (95% confidence interval, 0.68–0.99; *P* < 0.0001), again with the sensitivity and specificity values being 100% and 78%, respectively (Fig. 5d). The AUC values for agmatine and spermidine were at or under 0.8 (data not shown).

#### Correlations between brain and blood arginine metabolites

Because the blood and brain tissue were collected from the same animals, correlational analyses were conducted to determine whether neurochemical variables in the blood correlated with those in each brain region in both WT and Tg mice at both ages. No significant correlations were identified (data not shown).

## Discussion

APPswe/PS1ΔE9 transgenic mice develop age-related Aβ accumulation and plaque formation beginning from 4 months of age, and memory deficits that become progressively evident as the animals age^33^. The present study assessed behavioural performance in both the reference and working memory versions of the water maze task, and determined whether there were associated changes in the arginine metabolic profile either in the brain or plasma samples of Tg mice at 7 and 13 months of age. While impaired behavioural performance and altered brain arginine metabolism both became more prominent in Tg mice at 13 months of age, the plasma profile of arginine metabolism, amines in particular, was most prominently altered at 7 months of age.

### Age-related behavioural impairments in APP/PS1 mice

We tested water maze learning in both cued and spatial navigation tasks. The 7-month-old Tg mice showed no impairment in the visually cued navigation task, nor in either the working or in reference spatial memory tasks. In contrast, the 13-month-old Tg animals were clearly impaired in the spatial navigation tasks, although not in the cued version. These older Tg mice also showed a greater degree of thigmotaxic swimming relative to their age-matched WT littermates, indicating the use of a non-spatial learning strategy to locate the platform. However whether thigmotaxis impedes the use of a spatial strategy or results from a failed spatial strategy is unclear. Previously, other studies have identified that increased thigmotaxis in the water maze of APPswe/PS1ΔE9 mice can be reversed by therapy^61,62^. Thus, our results, demonstrating a clear age-associated performance impairments in the water maze task in Tg mice, are consistent with earlier reports of impaired water maze performance at 18, but not 6, months of age when widespread Aβ deposition is seen in the brain^35^.

The impairment of Tg mice in the working memory version of the water maze task is consistent with the deficit reported in a working memory version of the radial arm water maze task in the same strain of APPswe/PS1ΔE9 mice^35^. Similarly, rats with intracerebroventricular infusion of pre-aggregated Aβ_25–35_ were impaired preferentially in the working memory tasks when compared to the reference memory tasks^40,63–67^. Together, these data indicate that working memory performance can be a sensitive indicator of cognitive function in animal models of AD.

### Altered brain arginine metabolism with age in APP/PS1 mice

The semi-essential amino acid L-arginine can be metabolised to form a number of bioactive molecules that are essential in maintaining the normal function of the nervous system. Recent research using human post-mortem brain tissue has demonstrated an altered metabolic profile of L-arginine, including its downstream polyamines, in AD^6,8^. Experimentally, a single central administration of pre-aggregated Aβ_25–35_ peptide leads to impaired behavioural performance and altered L-arginine metabolic profile in the brain, and the latter appears to be associated with the degree of cognitive impairment^29,40^. Moreover, there are also altered brain arginine metabolic profiles and cognitive deficits with age in rats, as part of the normal ageing process^44,47,48,50,52,68–70^. The present study obtained for the first time the brain arginine metabolic profiles in 7- and 13-month-old male APP/PS1 mice and their age- and sex-matched littermates. Given the proposed causative role of Aβ in AD^1,3^, it is of interest to compare the profile pattern changes from these Aβ mice with that from AD patients.

At the 7-month-age point when APP/PS1 mice were largely unimpaired in the water maze tasks, there were virtually no genotype differences in other neurochemical variables determined in the prefrontal cortex, hippocampus, parahippocampal region and cerebellum, apart from mild changes in agmatine, spermine and arginase I protein expression. These findings suggest that brain arginine metabolism is affected only mildly during this early stage of Aβ accumulation in the brain.

At the 13-month-age point when APP/PS1 mice displayed prominent behavioural deficits, more neurochemical changes were evident in a neurochemical- and region-specific manner, but by and large these did not mirror the changes seen in the post-mortem AD brain tissue. Despite the marked AD- and age-related decreases in the total NOS activity and nNOS and eNOS protein levels in the human superior frontal gyrus and hippocampus^8^, for example, there were largely no genotype differences in these measures in the present study, except for increased nNOS expression in the Tg prefrontal cortex. While it has been shown that iNOS produces an excessive amount of NO in response to Aβ^22^, iNOS activity or protein expression was not detectable in Tg mice under the present experimental conditions. Alternative approaches, including immunohistochemistry, may need to be used in the future to determine whether iNOS changes with age in the brains of APPswe/PS1ΔE9 mice. Arginase competes with NOS for the substrate L-arginine, and is also one of the key enzymes in the urea cycle^71^. While there are AD-related increases in arginase activity and arginase II protein expression in the human hippocampus and superior frontal cortex^8^, these measurements were largely unaffected in APP/PS1 mice at 13 months of age, except for reduced arginase activity in the Tg parahippocampal region.

For L-arginine and its four downstream amino acids, the major changes observed were increased L-arginine and L-ornithine levels in APP/PS1 mice at 13 months of age. While a link between arginine deprivation and AD has been proposed^72^, our results clearly indicate no deprivation of free L-arginine in the mouse brain with long-term Aβ accumulation and deposition. Our findings of increased L-ornithine levels in the prefrontal cortex, hippocampus and parahippocampal region may suggest a shift of arginine metabolism towards the arginase pathway, perhaps in response to the high load of Aβ deposition in the brain. However, the general lack of genotype effects on arginase enzyme activity and protein expression does not support this straight-forward relationship. L-ornithine can be metabolised to produce glutamate and GABA^12^, and we did not see genotype differences in either measurement. Interestingly, González-Domínguez et al. reported altered glutamate levels in the hippocampus, cortex and cerebellum in an APPswe/PS1ΔE9 strain at 6 months of age^73^. It is currently unclear whether the differences in animals’ genetic background, age of analysis and/or behavioural experience between the two studies contribute to the discrepancy.

Regarding the four amines, we found decreased agmatine levels in the hippocampus and increased putrescine and spermine levels in the prefrontal cortex in 13-month APP/PS1 mice, which contrast with the higher level of agmatine in the parahippocampal region, but are consistent with higher spermine level in the prefrontal cortex, in 7-month Tg mice. Agmatine is decarboxylated arginine that serves as an alternative precursor of polyamines, regulates the production of NO and polyamines, interacts with multiple receptor subtypes and participates directly in learning and memory processes^12,25,27^. Hence, altered agmatine levels may have significant influences on other metabolic pathways of L-arginine, receptor function and learning and memory ability. On the basis of the pharmacological roles of agmatine, we speculate that the increased agmatine in young Tg mice might be a protective mechanism in response to the initial accumulation of Aβ and/or Aβ-associated pathologies, and that decreased levels at 13 months of age might represent the inability of the brain to combat with high loads of Aβ. It is of interest to emphasise the increased spermine levels in the prefrontal cortex of Tg mice at both ages with a greater effect at older age (the present study) and in the frontal cortex of AD patients^6^. In a recent preliminary study, interestingly, we observed that the levels of polyamines spermidine and spermine were markedly increased in the prefrontal cortex, hippocampus and parahippocampal region of 17-month APP/PS1 mice, again with the strongest effect in the prefrontal cortex. Because the prefrontal cortex has early amyloid deposition in APPswe/PS1ΔE9 mice^38^ and spermidine and spermine have an excitatory effect on the NMDA receptors^74^, it is possible that excitotoxicity may originate in the prefrontal cortex of APPswe/PS1ΔE9 mice and develop from there with age. The long-lasting changes in polyamines in the brain may be associated with age-related increases in amyloid load.

### Age- and genotype-related changes in plasma arginine metabolism

For prognostic detection of AD, it would be beneficial to have non-invasive biological markers of incipient disease prior to pathological decline in order to maximise the opportunity for therapeutic intervention. Blood samples are increasingly becoming routine biomarkers for disease/pathologic staging and organ status due to the possibility of repeated non-invasive sampling. In the present study, we found increased plasma levels of L-arginine, L-citrulline, L-ornithine, glutamate and GABA with age regardless of genotype, indicating that the changes of these amino acids are part of the normal aging process. Theoretically, the concentrations of L-arginine and its metabolites in the plasma should represent the balance of their production, uptake and clearance. The small intestine releases large amounts of L-citrulline into the circulation, which is then be taken up by kidneys to generate L-arginine^75,76^. Hence, the functional status of these peripheral organs would influence the plasma concentrations of L-citrulline and L-arginine. The lack of significant correlations for the five amino acids between the plasma and brain tissue samples suggest that the brain is not the major source for these amino acids in the plasma. It should be noted that the plasma samples were obtained from the trunk blood. It is currently unclear whether the venous and arterial plasma would have different profiles.

It is of interest to emphasise that the levels of plasma amines changed in APP/PS1 mice at 7 months of age, which is the age when few genotype-related changes in behaviour or brain arginine metabolism were observed. As for the five amino acids, we failed to detect significant correlations for agmatine or the three polyamines between the plasma and brain tissue samples in same animals. Despite the complicated issue regarding the sources of the four amines, the observed changes in plasma agmatine and polyamines may reflect the responses of the peripheral organs associated with their production and clearance of the initial accumulation of Aβ. Hence, they may potentially be early markers for peripheral Aβ pathology. A number of studies have reported alterations in other metabolic pathways in the serum and peripheral organs, such as liver, kidney, spleen and thymus at 6 months of age in APPswe/PS1ΔE9 mice^77–79^. Since there were no significant correlations in L-arginine metabolome between the plasma and brain tissue samples, and the time course of change was markedly different for plasma vs. brain samples, the peripheral organs and the brain may respond differently to heightened Aβ pathology in APP/PS1 mice. It will be interesting to investigate more systematically in both animals and humans the time course of alterations in the plasma arginine metabolic profile to build upon the present findings.

The ROC curve analysis, a fundamental diagnostic evaluation tool in medicine^80^, indicated that the possibility of lower levels of putrescine and spermine in APP/PS1 mice relative to their WT littermates was as high as over 90%. Moreover, the sensitivity and specificity of the putrescine and spermine measurements between APP/PS1 and WT mice were found to be 100% and 78%, respectively. These findings suggest that the plasma level of putrescine or spermine could be used to detect animals with Aβ accumulation at an age when there are no or only very mild behavioural deficits and neurochemical changes in the brain.

## Conclusions

APPswe/PS1ΔE9 mice display an age-dependent accumulation of Aβ in the brain^32,39^, and hence serve as a useful experimental tool for understanding the role of Aβ in the development of AD-like pathologies. The present study, for the first time, demonstrated age-related cognitive deficits and alterations in brain arginine metabolism in a neurochemical- and region-specific manner in this Aβ model. Because the brain tissues collected from both hemispheres were used for the enzyme assays, western blot, HPLC and LC/MS assays, we were unable to quantify the Aβ load in APP/PS1 mice, hence no correlational analysis with brain Aβ was performed. It is of interest to note that the overall pattern of the metabolic profile changes in APP/PS1 mice differs from that obtained from the post-mortem brain tissue of AD patients^8^, although the experimental protocols and procedures used in both studies are the same. The discrepancy could be due to the animal model used, because the APP/PS1 mouse model only recapitulates Aβ pathology (with little tau pathology or neuronal loss), and hence mainly reflects the early stage of AD. It would be worth investigating whether late time points of the Aβ model or other types of models would better replicate the arginine metabolic profile changes seen in the brains of AD patient at late stage^8^. It should be also noted that the pattern of changes in the brain arginine metabolic profile in APP/PS1 mice was different from that seen in animals with a single intracerebroventricular infusion of Aβ_25–35_^29,30^, indicating differential responses of the central nervous system to a sudden increase vs. graduated accumulation of Aβ.

The present study also demonstrates genotype-specific alterations in plasma agmatine and polyamines at 7 months of age that represents an early stage of brain Aβ deposition. Although APP/PS1 mice at this age point displayed very mild changes in behavioural function and brain arginine metabolism, our ROC analysis data clearly indicated the possibility of plasma putrescine or spermine in detecting animals with Aβ accumulation. To this end, the plasma results merit further investigation in order to explore their potential for the early detection of AD. Graham et al. analysed the blood metabolome of cases of mild cognitive impairment (MCI), who subsequently developed AD, vs. healthy age-matched controls^81^. L-arginine metabolism and polyamine metabolism (two interlinked areas of metabolism, referred to as the broad L-arginine metabolome in the present study) were differentially disrupted in this well-defined clinical cohort. These arginine-centric markers could be used to predict MCI patients ‘at risk’ of developing AD, up to 2 years earlier than a conventional clinical diagnosis^81^. Moreover, Gratieri et al.^82^ measured the plasma concentrations of the polyamines spermidine and spermine in control, MCI and late-onset AD (LOAD) cases (some MCI cases had converted to AD after 4 years of clinical follow-up). Polyamines could distinguish LOAD from the MCI group, and spermine in particular could predict the conversion of MCI to LOAD^82^. Further studies of large human cohorts will be required to validate whether altered arginine metabolism (polyamines in particular) in blood could be used as a reliable biomarker for predicting the development, and monitoring the progression, of AD.

## Electronic supplementary material

Supplementary Figure 1

Supplementary Figure 2

Supplementary Figure Legend
