# Supplementary figures and images for: Altered plasma arginine metabolome precedes behavioural and brain arginine metabolomic profile changes in the APPswe/PS1ΔE9 mouse model of Alzheimer’s disease
Source: Transl Psychiatry. 2018 May 25;8:108. doi: 10.1038/s41398-018-0149-z (PMC5970225; doi:10.1038/s41398-018-0149-z)

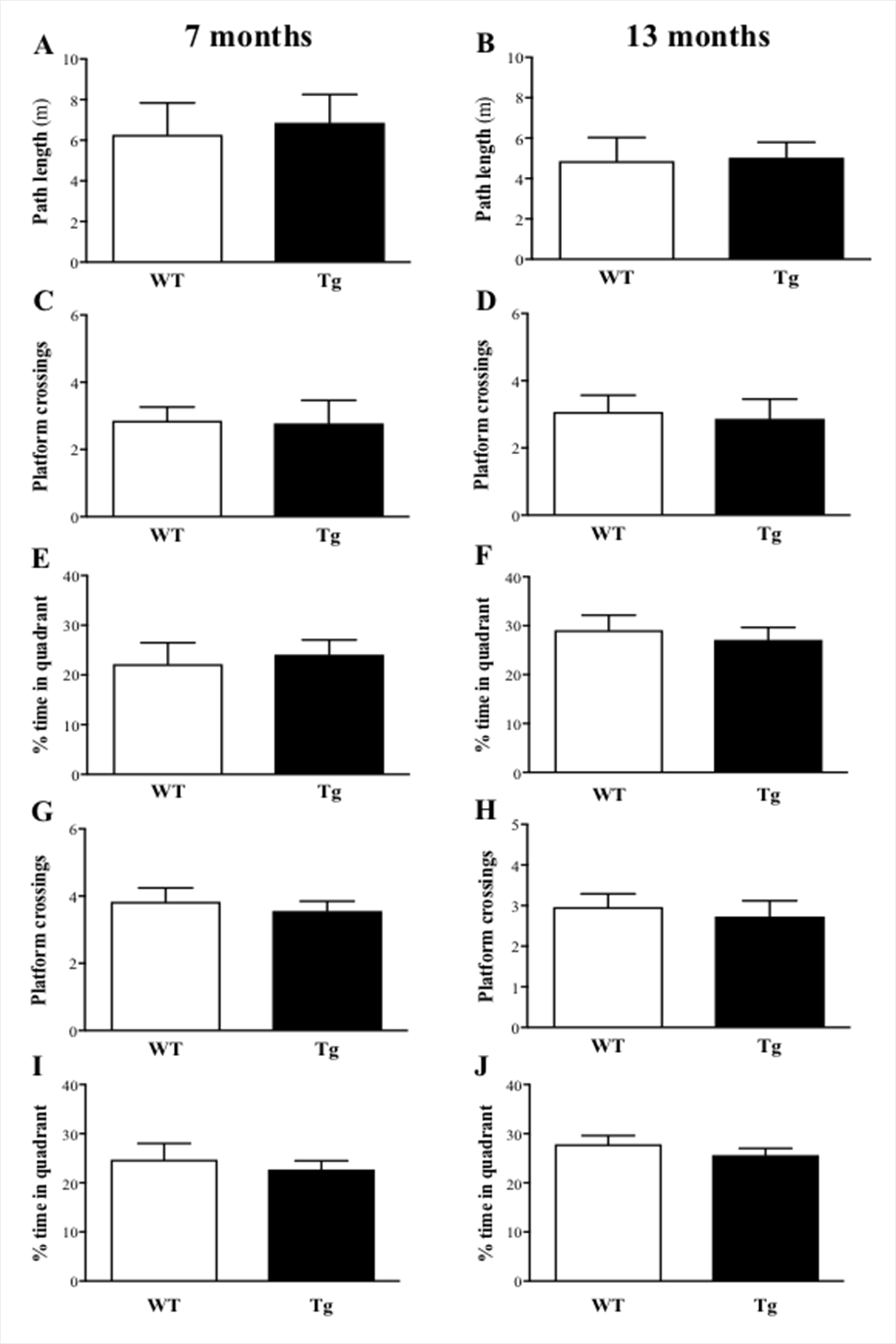

Supplement: Supplementary file 1 — Supplementary Figure 1 [file 41398_2018_149_MOESM1_ESM.tif]

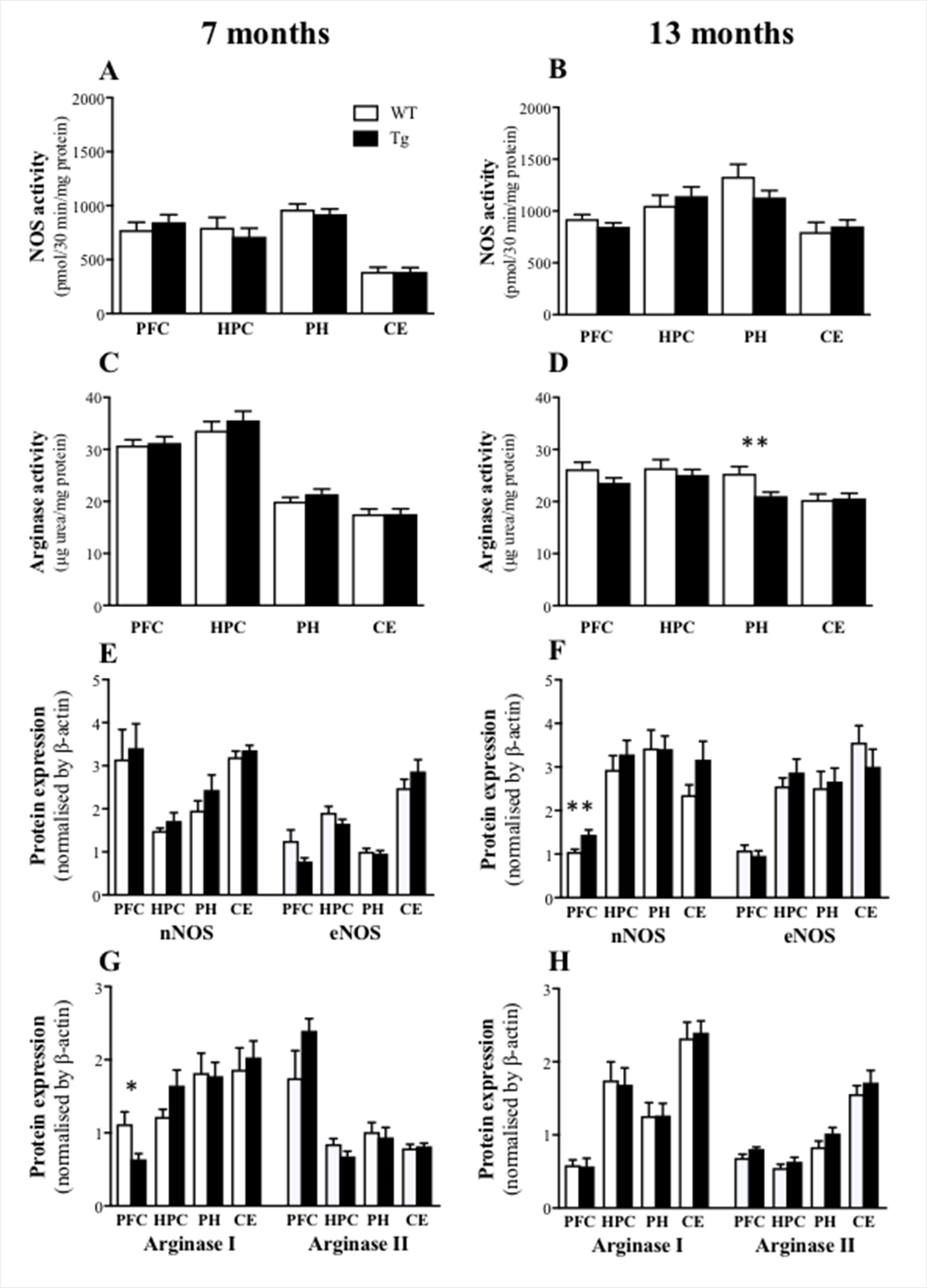

Supplement: Supplementary file 2 — Supplementary Figure 2 [file 41398_2018_149_MOESM2_ESM.tif]
