# Supplementary material for: Altered plasma arginine metabolome precedes behavioural and brain arginine metabolomic profile changes in the APPswe/PS1ΔE9 mouse model of Alzheimer’s disease
Source: Transl Psychiatry. 2018 May 25;8:108. doi: 10.1038/s41398-018-0149-z (PMC5970225; doi:10.1038/s41398-018-0149-z)
Supplement: Supplementary file 3 — Supplementary Figure Legend [file 41398_2018_149_MOESM3_ESM.docx]

**Supplementary Figure 1**

Animals’ performance in the probe tests of the reference memory (A – F) and working memory (G – J) versions of the water maze task. Mean (± SEM) path length to the platform (A and B), number of platform crossings (C and D) and percentage of time in the target quadrant (E and F) in the working memory version, and platform crossings (G and H) and percentage of time in the target quadrant (I and J) in the working memory version, in the wild-type (WT) and APPswe/PS1ΔE9 transgenic (Tg) mice at 7 (A, C, E, G and I) and 13 (B, D, F, H and J) months of age. There was no significant difference between groups for each measurement at either age point.

**Supplementary Figure 2**

Mean (± SEM) activity of NOS (A and B) and arginase (C and D), and protein expression of neuronal and endothelial NOS (nNOS and eNOS respectively; E and F) and arginase I and arginase II (G and H) in the prefrontal cortex (PFC), hippocampus (HPC), parahippocampal region (PH) and cerebellum (CE) of the wild-type (WT) and APPswe/PS1ΔE9 transgenic (Tg) mice at 7 (A, C, E and G) and 13 (B, D, F and H) months of age. There was reduced arginase I protein level expression in the PFC in Tg mice at 7 months of age. Tg mice at 13 months of age had reduced arginase activity in the PH and increased nNOS protein levels in the PFC relative to the WT mice. * indicates significant difference between genotypes at **p* < 0.05, ** *p* < 0.01.
